# Impact of age on mortality and transfer to long-term care in patients in an intensive care unit

**DOI:** 10.1186/s12877-023-04526-5

**Published:** 2023-12-12

**Authors:** Eunki Chung, Kyung Soo Chung, Ah Young Leem, Ala Woo, Moo Suk Park, Young Sam Kim, Su Hwan Lee

**Affiliations:** Division of Pulmonary and Critical Care Medicine, Department of Internal Medicine, Severance Hospital, Yonsei University College of Medicine, 50-1 Yonsei-ro, Seodaemun-gu, Seoul, 03722 Republic of Korea

**Keywords:** Age, Intensive care unit, Mortality, Prognosis

## Abstract

**Background:**

In the global trend of population aging, age is one of the significant factors to be considered in critically ill patients. However, the impact of age on clinical outcomes and long-term prognosis in this population varies across different studies.

**Methods:**

We conducted a retrospective cohort analysis for patients admitted to the medical intensive care unit (ICU) (30 beds) between January 2017 and December 2020 at the tertiary referral hospital in Korea. Patients were classified into three groups according to age: <65 years, old age (65–79 years), and very old age (≥ 80 years). Subsequently, enrolled patients were analyzed for acute mortality and long-term prognosis.

**Results:**

Among the 1584 patients, the median age was 67.0 (57.0–76.0) years, and 65.2% were male. Median ICU length of stay (LOS) (8, 9, and 10 days in < 65, 65–79, and ≥ 80 years, respectively; *p* = 0.006) and the proportion of patients who were transferred to long-term care hospital at the time of discharge (12.9% vs. 28.3% vs. 39.4%, respectively; *p* < 0.001) increased with age. Multivariable logistic analysis showed no significant difference in the 28-day mortality in the old age (adjusted odds ratio [aOR] 0.88; 95% confidence interval [CI] 0.65–1.17) and very old age (aOR 1.05; 95% CI 0.71–1.55) groups compared to that in patients with age < 65 years. However, the relevance of the proportion of ICU LOS ≥ 7 days and transfers to other hospitals after discharge increased with age.

**Conclusions:**

Age did not affect acute mortality in critical illness patients. However, surviving older age groups required more long-term care facilities compared to patients younger than 65 years after acute management. These results indicate that in an aging society, the importance of not only acute management but also long-term care facilities may increase for critical illness patients.

**Supplementary Information:**

The online version contains supplementary material available at 10.1186/s12877-023-04526-5.

## Background

The allocation and utilization of limited intensive care unit (ICU) resources has been a significant ongoing health care issue [1], and has gained increased attention due to the exacerbated shortage of critical care facilities globally throughout the COVID-19 era [2, 3]. Various clinical perspectives, such as acute illness, the patient’s medical history, and age determines which patients should be prioritize for ICU hospitalization.

However, controversy exists regarding the consideration of age as a basis for ICU admission [1]. Previous research suggests that the ICU mortality rate is higher among the older population [4, 5], and from a perspective based on life expectancy, age could be considered a criterion for admission [2, 6]. Nevertheless, research indicating greater benefits of ICU admission in the older population and a favorable long-term prognosis after ICU admission in functional older patients provides evidence that relying solely on age as a criterion for admission may be insufficient [5, 7]. Furthermore, the absolute number and proportion of the older population are continuously increasing worldwide [8]. Consequently, the population of older patients admitted to the ICU is also increasing [9, 10], and it is essential to consider the prioritization of older patients’ ICU admission in the allocation of critical care resources.

Therefore, this study was aimed at comparing the clinical course and long-term prognosis after ICU admission among different age groups to determine whether age should be one of the criteria for allocating ICU for older patients on hospitalization.

## Methods

### Study design and population

This single-center, retrospective cohort study was conducted at a tertiary hospital in South Korea, comprising a medical ICU with 30 beds. South Korea demographically became an aging society in 2017, and due to the COVID-19 pandemic in 2021, the research period was set between January 2017 and December 2020, which included all medical patients who were admitted to the ICU [11]. All 1584 patients admitted to the medical ICU during that period were included in the study. Based on the commonly used definitions of old age and very old age [4, 12, 13], the age classification criteria were set at 65 and 80 years, respectively. Subsequently, the patients were categorized into three age groups: <65 years, old age (65–79 years), and very old age (≥ 80 years) and were compared accordingly.

### Data collection and definition

At the time of ICU admission, demographic features, vital signs, the Charlson Comorbidity Index (CCI), the Sequential Organ Failure Assessment (SOFA) score, and laboratory data were collected and investigated. In addition, information regarding endotracheal intubation, use of facility before admission, year of ICU admission, reason for ICU admission, ICU length of stay (LOS), general ward LOS before and after ICU discharge, 28-day and 90-day mortality after ICU admission, DNR status after ICU admission, and discharge destination were also investigated. The assessment of physical function before and after ICU admission was conducted using the ICU Mobility Scale (IMS) [14]. The primary outcome included 28-day mortality after ICU admission, whereas the secondary outcomes included 90-day mortality after ICU admission, ICU LOS ≥ 7 days, and transfer to another medical facility at the time of discharge.

### Statistical analysis

For comparing different age groups, Pearson’s chi-square test was used to compare categorical variables. For continuous variables, normality was tested using the Shapiro–Wilk test, and Kruskal–Wallis test was employed for non-normal distributions. The comparison of paired non-parametric groups was conducted using the Wilcoxon signed-rank test. Trends in non-parametric data with three or more groups were confirmed using the Jonckheere-Terpstra test. Covariates were established based on clinical knowledge for sex, BMI, CCI, SOFA, use of facility before admission, general ward LOS before ICU admission, and DNR status after ICU admission. Subsequently, using the covariates as references, adjusted odds ratios (aOR) for the primary and secondary outcomes were calculated for each age group through multivariable logistic regression analysis. In the overall analysis, p-values < 0.05 were considered statistically significant. Statistical Package For The Social Sciences (v.26.0; Armonk, New York, USA) and R software (v.4.2.1; R Foundation for Statistical Computing, Vienna, Austria) were used for the statistical analysis.

## Results

### Baseline characteristics

**Fig. 1:**
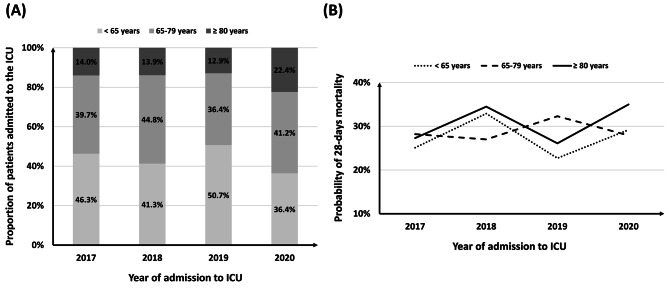
ICU admission and mortality by age group, stratified by year of admission. **(A)** ICU admission rates by age group for each year. **(B)** 28-day mortality rates by age group for each year. ICU, intensive care unit

A total of 1584 patients were included in the analysis. The median age was 67.0 (57.0–76.0), and 65.2% of participants were male (Table 1). Over the entire 4-year period, the proportion of very old patients was 15.6%. From 2017 to 2019, the admission rate of very old ICU patients accounted for 10% of the total patients, but in 2020, it sharply increased to 22.4% in Fig. 1A (Standardized Jonckheere-Terpstra test statistic = 2.4, *p* = 0.015). The changes in 28-day mortality rates by age group according to the year of ICU admission are depicted in Fig. 1B. Although fluctuations were observed across different years, no significant trend was observed (Standardized Jonckheere-Terpstra test statistic = 1.7, *p* = 0.082). The predicted mortality rate corresponding to the median SOFA score upon admission was 33% [15], and endotracheal intubation was performed in 74.7% of all ICU admitted patients. The proportion of patients with a CCI score ≥ 5 was 28.8%, and the median CCI was 3.0 (2.0–5.0), indicating a high overall comorbidity burden among patients admitted to the ICU. The IMS at ICU discharge were statistically significantly higher than at ICU admission (*p* < 0.001). Nearly a quarter of the patients (28.3%) died within 28 days, and among those who survived, 71.4% were discharged.

**Table 1:** Baseline characteristics of participants

Characteristics	Total participants (N = 1584)	< 65 years (N = 694)	65–79 years (N = 643)	≥ 80 years (N = 247)	*p*-value
**Age (years)**	67.0 (57.0–76.0)	55.0 (47.0–61.0)	73.0 (69.0–76.0)	83.0 (81.0–86.0)	< 0.001
**Sex, no (%)**					0.347
Male, no.	1032 (65.2)	464 (66.9)	415 (64.5)	153 (61.9)	
Female, no.	552 (34.8)	230 (33.1)	228 (35.5)	94 (38.1)	
**Mean arterial pressure (mmHg)**	84.0 (72.0–97.0)	86.0 (72.0–98.0)	84.0 (72.0–97.0)	82.0 (71.0–94.0)	0.026
**Heart rate (/min)**	99.5 (82.0–117.0)	101.0 (85.0–118.0)	98.0 (81.0–119.0)	95.0 (77.0–113.0)	0.008
**Respiratory rate (/min)**	21.0 (18.0–25.0)	22.0 (18.0–25.0)	21.0 (18.0–25.0)	20.0 (16.0–24.0)	< 0.001
**BMI (kg/m** ^**2**^ **)**	22.3 (19.5–25.1)	22.1 (19.2–25.1)	22.5 (20.0–25.3)	22.1 (19.4–24.6)	0.072
**Use of facility before admission, no (%)**	315 (19.9)	138 (19.9)	131 (20.4)	46 (18.6)	0.842
**ICU admission year, no (%)**					< 0.001
2017	473 (29.9)	219 (31.6)	188 (29.2)	66 (26.7)	
2018	397 (25.1)	164 (23.6)	178 (27.7)	55 (22.3)	
2019	357 (22.5)	181 (26.1)	130 (20.2)	46 (18.6)	
2020	357 (22.5)	130 (18.7)	147 (22.9)	80 (32.4)	
**CCI**	3.0 (2.0–5.0)	3.0 (2.0–4.0)	3.0 (2.0–5.0)	3.0 (2.0–5.0)	< 0.001
**CCI, no (%)**					< 0.001
0–1	312 (19.7)	170 (24.5)	97 (15.1)	45 (18.2)	
2–4	816 (51.5)	355 (51.2)	334 (51.9)	127 (51.4)	
≥ 5	456 (28.8)	169 (24.4)	212 (33.0)	75 (30.4)	
**Comorbidities, no (%)**					
HTN	910 (57.4)	265 (38.2)	454 (70.6)	191 (77.3)	< 0.001
DM	600 (37.9)	210 (30.3)	274 (42.6)	116 (47.0)	< 0.001
CKD	408 (25.8)	126 (18.2)	187 (29.1)	95 (38.5)	< 0.001
Cardiovascular disease	312 (19.7)	70 (10.1)	160 (24.9)	82 (33.2)	< 0.001
Cerebrovascular disease	201 (12.7)	42 (6.1)	103 (16.0)	56 (22.7)	< 0.001
Liver disease	262 (16.5)	154 (22.2)	91 (14.2)	17 (6.9)	< 0.001
Asthma	91 (5.7)	30 (4.3)	41 (6.4)	20 (8.1)	0.061
COPD	156 (9.8)	53 (7.6)	86 (13.4)	17 (6.9)	< 0.001
Solid cancer	466 (29.4)	176 (25.4)	232 (36.1)	58 (23.5)	< 0.001
Psychological disorder	32 (2.0)	20 (2.9)	11 (1.7)	1 (0.4)	0.046
Dementia	69 (4.4)	5 (0.7)	35 (5.4)	29 (11.7)	< 0.001
**Reason for ICU admission**					0.017
Cardiogenic cause	53 (3.3)	21 (3.0)	26 (4.0)	6 (2.4)	
Respiratory cause	917 (57.9)	393 (56.6)	374 (58.2)	150 (60.7)	
Gastrointestinal cause	227 (14.3)	119 (17.1)	82 (12.8)	26 (10.5)	
Nephrogenic cause	108 (6.8)	47 (6.8)	45 (7.0)	16 (6.5)	
Infectious cause	256 (16.2)	98 (14.1)	109 (17.0)	49 (19.8)	
Hematologic cause	23 (1.5)	16 (2.3)	7 (1.1)	0 (0.0)	
**ICU LOS (day)**	9.0 (4.0–18.0)	8.0 (4.0–16.0)	9.0 (5.0–19.0)	10.0 (6.0–19.0)	0.006
**General ward LOS before ICU admission (day)**	2.0 (0.0–13.0)	2.0 (0.0–15.0)	2.0 (0.0–12.0)	1.0 (0.0–11.0)	0.066
**General ward LOS after ICU discharge (day)**	12.0 (0.0–38.0)	13.0 (0.0–46.0)	13.0 (0.0–37.0)	10.0 (0.0–25.0)	0.008
**IMS on ICU admission**	0.0 (0.0–0.0)	0.0 (0.0–0.0)	0.0 (0.0–0.0)	0.0 (0.0–0.0)	0.029
**IMS on ICU discharge**	0.0 (0.0–1.0)	0.0 (0.0–2.0)	0.0 (0.0–1.0)	0.0 (0.0–1.0)	0.014
**DNR status after ICU admission, no (%)**	585 (36.9)	230 (33.1)	258 (40.1)	97 (39.3)	0.022
**28-day mortality, no (%)**	449 (28.3)	188 (27.1)	184 (28.6)	77 (31.2)	0.464
**90-day mortality, no (%)**	614 (38.8)	244 (35.2)	268 (41.7)	102 (41.3)	0.034
Discharge destination*					< 0.001
Home	633 (71.4)	334 (81.5)	228 (67.3)	71 (51.8)	
General hospital	50 (5.6)	23 (5.6)	15 (4.4)	12 (8.8)	
Long–term care hospital	203 (22.9)	53 (12.9)	96 (28.3)	54 (39.4)	
**Intubation, no (%)**	1184 (74.7)	509 (73.3)	483 (75.1)	192 (77.7)	0.379
**SOFA**	9.0 (6.0–12.0)	8.0 (5.0–11.0)	9.0 (6.0–12.0)	9.0 (7.0–12.0)	0.009
**Laboratory data**					
White blood cell (10^3^/µL)	12.1 (7.5–18.3)	10.9 (6.5–16.7)	12.9 (8.1–19.2)	13.5 (8.6–19.6)	< 0.001
Albumin (g/dL)	2.7 (2.4–3.0)	2.7 (2.4–3.1)	2.7 (2.3–3.0)	2.6 (2.3–2.9)	< 0.001
Blood urea nitrogen	31.0 (19.2–50.1)	27.1 (15.5–45.8)	31.4 (20.9–50.8)	39.4 (26.8–59.0)	< 0.001
Creatine	1.1 (0.6–1.8)	0.9 (0.6–1.8)	1.1 (0.7–1.8)	1.3 (0.9–2.0)	< 0.001
C-reactive protein (mg/L)	91.8 (34.0–185.6)	74.8 (24.6–171.0)	101.6 (43.2–189.1)	120.6 (45.8–219.8)	< 0.001
Delta neutrophil index	2.6 (0.9–7.7)	2.7 (0.8–7.6)	2.5 (1.0–7.9)	2.5 (0.6–7.2)	0.768

* 886 patients survived and were discharged from the hospital

BMI, body mass index; CCI, Charlson Comorbidity index; ICU, intensive care unit; LOS, length of stay; IMS, ICU Mobility Scale; DNR, do not resuscitate; SOFA, sequential organ failure assessment score; HTN, hypertension; DM, diabetes mellitus; CKD, chronic kidney disease; COPD, chronic obstructive pulmonary disease

**Fig. 2:**
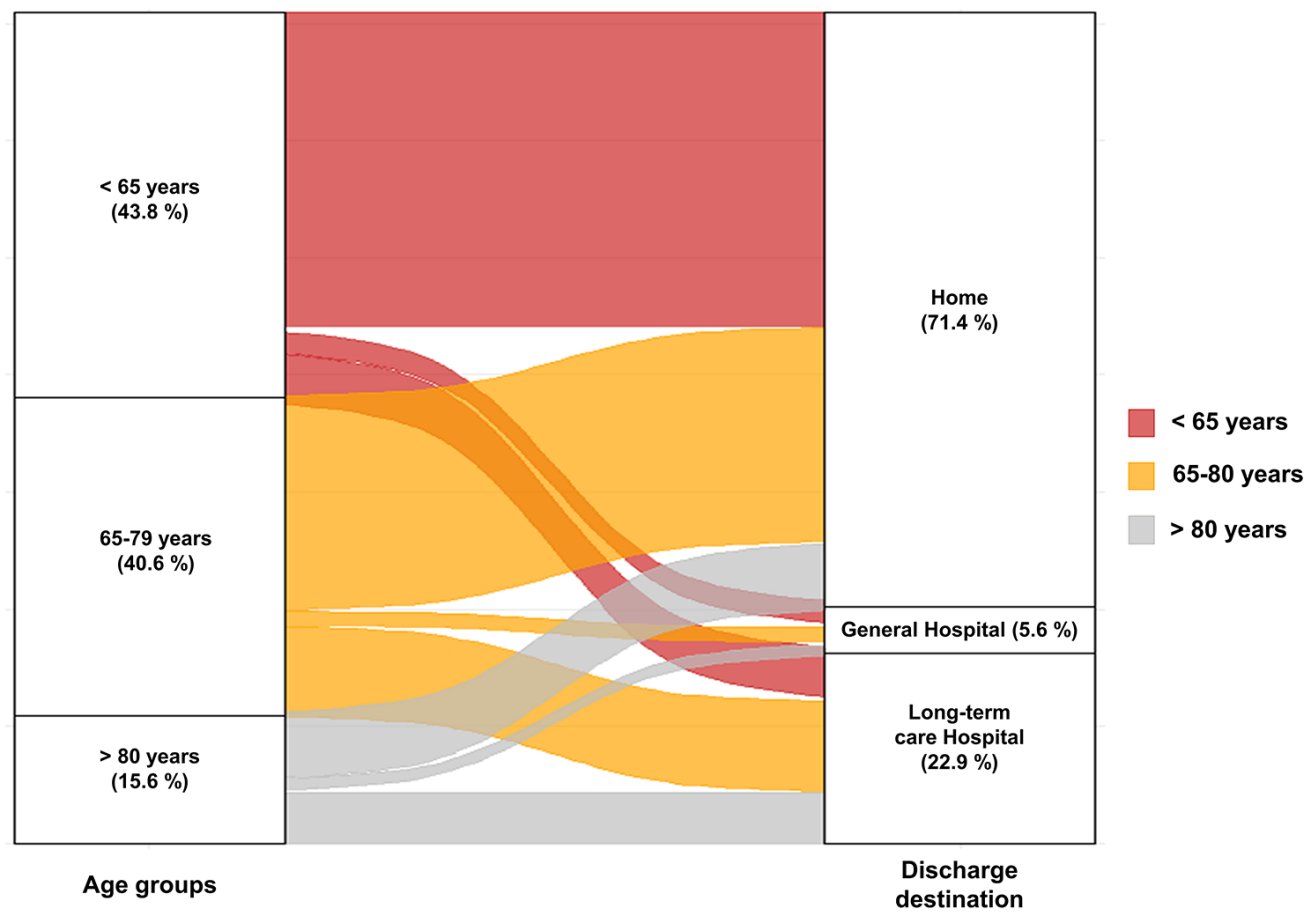
Status of discharge destinations by age group

The proportion of patients with a CCI score ≤ 1 was lower in old and very old patients compared to those aged < 65 (24.5% in < 65 years, 15.1% in 65–79 years, 18.2% in ≥ 80 years; *p* < 0.001). Additionally, the median SOFA score was higher in old age and very old age (8.0 vs. 9.0 vs. 9.0; *p* = 0.009), indicating a higher frequency of comorbidities and poorer overall condition among the old and very old patients compared to the younger patients. As age increased, there was an increasing trend in the median ICU LOS (8.0 days vs. 9.0 days vs. 10.0 days; *p* = 0.006) and the proportion of transfers to long-term care hospital at the time of discharge (12.9% vs. 28.3% vs. 39.4%, *p* < 0.001). Conversely, the rate of discharge to home decreases as the age group increases, as depicted in Fig. 2. However, there were no statistically significant differences in the 28-day mortality and the use of facility before admission among the different age groups.

### Multivariable analysis for primary and secondary outcomes

The odds ratios for each primary and secondary outcome across the different age groups are presented in Table 2. The results of the adjusted multivariable analysis for both 28- and 90-day mortality showed no significant age group differences. Only the very old patients (aOR 1.67; 95% CI 1.22–1.29) exhibited a higher association with ICU LOS ≥ 7 days compared to the patients aged < 65 and 65–79 years. As age groups get older, the aOR values for discharge to a long-term care hospital increase (aOR 4.27; 95% CI 2.64–6.88, ≥ 80 years), while the aOR values for discharge to home decreases (aOR 0.24; 95% CI 0.15–0.38, ≥ 80 years).

**Table 2:** Logistic regression analysis for primary and secondary outcomes

Age groups	< 65 years		65–79 years		≥ 80 years	
	95% CI	*p*-value	95% CI	*p*-value	95% CI	*p*-value
28-days mortality, no (%)	188 / 694 (27.1)		184 / 643 (28.6)		77 / 247 (31.2)	
OR	1.0 (ref)		1.08 (0.85–1.37)	0.534	1.22 (0.89–1.67)	0.221
aOR*	1.0 (ref)		0.88 (0.65–1.17)	0.367	1.05 (0.71–1.55)	0.804
90-days mortality, no (%)	244 / 694 (35.2)		268 / 643 (41.7)		102 / 247 (41.3)	
OR	1.0 (ref)		1.32 (1.06–1.64)	0.014	1.30 (0.96–1.75)	0.086
aOR*	1.0 (ref)		1.09 (0.80–1.49)	0.586	1.10 (0.73–1.66)	0.658
ICU LOS ≥ 7 days	398 / 694 (57.3)		402 / 643 (62.5)		171 / 247 (69.2)	
OR	1.0 (ref)		1.24 (1.00–1.55)	0.054	1.67 (1.23–2.28)	0.001
aOR*	1.0 (ref)		1.23 (0.98–1.53)	0.074	1.67 (1.22–1.29)	0.001
Discharge to home†	334 / 410 (81.5)		228 / 339 (67.3)		71 / 137 (51.8)	
OR	1.0 (ref)		0.47 (0.33–0.66)	< 0.001	0.25 (0.16–0.37)	< 0.001
aOR*	1.0 (ref)		0.47 (0.33–0.67)	< 0.001	0.24 (0.15–0.38)	< 0.001
Discharge to general hospital †	23 / 410 (5.6)		15 / 339 (4.4)		12 / 137 (8.8)	
OR	1.0 (ref)		0.78 (0.40–1.52)	0.463	1.62 (0.78–3.34)	0.196
aOR*	1.0 (ref)		0.76 (0.38–1.55)	0.454	1.48 (0.67–3.23)	0.330
Discharge to long–term care hospital †	53 / 410 (12.9)		96 / 339 (28.3)		54 / 137 (39.4)	
OR	1.0 (ref)		2.66 (1.83–3.86)	< 0.001	4.38 (2.80–6.86)	< 0.001
aOR*	1.0 (ref)		2.62 (1.78–3.86)	< 0.001	4.27 (2.64–6.88)	< 0.001

* Adjusted for sex, BMI, CCI, SOFA, use of facility before admission, general ward LOS before ICU admission, and DNR status after ICU admission

† 886 patients who survived

BMI, body mass index; CCI, Charlson comorbidity index; SOFA, sequential organ failure assessment score; aOR, adjusted odds ratio; CI, confidence interval

### Subgroup analysis according to the use of facility before admission

We conducted a subgroup analysis based on the use of facilities before admission, and the comparison of baseline characteristics is presented in Supplementary Table S1. In the group that used facilities before admission, BMI, albumin, and creatinine were significantly lower. However, ICU LOS, general ward LOS before and after ICU admission, and the rate of transferring to long-term care hospitals after discharge were higher in the group that used facilities before admission. We divided the groups based on the use of facilities before admission and conducted multivariable analysis, and the results were consistent with those of the overall participants (Supplementary Table S2–3).

## Discussion

This study investigated the association between age and ICU mortality rate, as well as the association between age and discharge to long-term care hospitals. No statistically significant difference was observed in the 28-day mortality among different age groups in this study. However, the ICU LOS and transfers to long-term care hospitals upon discharge increased with age.

The current study distinguishes itself from previous research by demonstrating that, among the old and very old age groups, there was no statistically significant difference in the 28- and 90-day mortality rates following ICU admission compared to patients aged < 65. Fuchs et al. reported that 28-day mortality increased with advancing age groups [4], and Mukhopadhyay et al. reported higher ICU mortality among old patients compared to those < 65. [16] However, Rai et al. reported that there was a notable decrease in mortality in very old patients in more recent ICU admissions. Additionally, there has been a significant increase in the proportion of patients being transferred to other hospitals upon discharge, particularly among the younger population [13]. This indicates that there is a decrease in mortality among the old and very old population with the advancement of medical care. Notably, an increasing proportion of patients require additional medical treatment after discharge, highlighting the need for further medical intervention beyond patients’ ICU stays. Our study results suggest that the ICU mortality rate is not higher among the old age population, and the increasing proportion of transfers to other hospitals upon discharge with advancing age aligns with the recent trend of ICU admissions. Therefore, in terms of mortality, it can be concluded that age alone is insufficient to be considered an independent criterion for ICU admission.

In other studies, researchers have focused on assessing the degree of frailty rather than age and used this information to predict post-ICU outcomes [17, 18]. The findings of this study, however, indicate that there was no significant difference in mortality rates across different age groups despite the more unfavorable admission status of old and very old patients. This underscores the potential necessity of considering additional factors, such as the effectiveness of ICU admission, alongside frailty. Table 1 suggests that higher CCI and SOFA scores indicated increased inflammatory markers, and lower albumin levels were observed in the old and very old age groups compared to the patients aged < 65. These findings indicate that overall health status upon admission is less important in the old and very old age groups compared to the younger age group. As there was no statistically significant difference in mortality among the age groups, possibly the benefits of ICU admission may be higher in the old and very old age groups. This aligns with the findings of Sprung et al., who reported an increasing effectiveness of ICU admission with increasing age. Moreover, their study suggested that the effectiveness of ICU treatment in older patients is greater in cases of lower severity than in cases of higher severity. This highlights the need for further research to determine the level of severity at which the effectiveness of ICU admission is maximized [7].

Another aspect that needs to be considered, in addition to mortality, is the healthcare cost. According to Karlsson et al., the cost per quality-adjusted life year increases more in older patients in ICU settings due to severe sepsis [19]. Studies on ICU costs have revealed that the greatest contributors to the overall expenses are mechanical ventilation and ICU LOS [20, 21]. Our study found a correlation that ICU LOS increases in higher age groups; therefore, although the costs were not further investigated in this study, it is possible that older age groups are associated with higher healthcare costs. Additionally, the result that the proportion of transfers to other hospitals increases with age further supports this hypothesis. In a study by Chappell et al., which reported that home care costs were significantly lower compared to long-term facility care [22], it was speculated that younger age groups, who have a higher likelihood of being discharged to their home, would consume relatively lower healthcare costs. These results are significant from an economic and policy standpoint.

Additionally, it is possible that the observed trend of decreasing LOS in general wards with increasing age after discharge from the ICU may be influenced by hospital policies. The investigated tertiary hospitals have a long-term care policy that recommends transferring patients to other long-term care hospitals or nursing homes who have completed acute treatment but cannot be discharged home and require long-term care. Therefore, old and very old patients with lower possibilities of being discharged home are transferred to another facility earlier than those aged < 65, which may result in shorter durations in general wards after ICU discharge. If the LOS in the subsequent hospital after transfer is also considered, the pattern of hospitalization duration in general wards may appear different.

One of the advantages of this study is that it demonstrates that old age, which was traditionally considered one of the factors influencing ICU mortality, has a low impact on mortality. This suggests that factors other than age should be considered a priority in ICU admissions. However, this study also had limitations. First, due to a single-center study, there may be regional biases in the sample, and therefore, validation through multicenter or national data may be necessary in the future. However, being a single-center study, it also has the advantage of reducing the impact of interventions, protocols, healthcare providers, and medical equipment on the results. Second, due to the retrospective nature of the study, we were unable to score the degree of frailty, known to be associated with worsening prognosis in ICU patients [17], and for the same reason, we did not attempt diverse evaluations of physical functions in ICU patients [23]. Lastly, the presence of selection bias due to DNR orders is a concern. In this study, we adjusted for the variable of DNR status after ICU admission as a covariate. However, it was not possible to aggregate cases where older patients who clinically required admission to the ICU had DNR orders and eventually passed away in general wards. This limitation may have resulted in the inclusion of only older patients with better ICU outcomes, potentially leading to a lack of differences in mortality rates among age groups. To address this issue, a large-scale analysis involving the entire population of deceased patients in the hospital will be needed in the future to determine the patient groups.

## Conclusions

There was no difference in the 28-day mortality based on age group among patients admitted to the ICU. However, this analysis revealed that older patients have a lower rate of returning home upon survival and are more likely to be transferred to long-term care hospitals. These findings have the potential to support informed decision-making in determining future ICU admissions.

### Electronic supplementary material

Below is the link to the electronic supplementary material.

Supplementary Material 1

## Data Availability

The datasets used or analyzed during the current study are available from the corresponding author on reasonable request.

## List of abbreviations

aOR: adjusted odds ratio
CCI: Charlson Comorbidity Index
CI: confidence interval
DNR: do-not-resuscitate
ICU: intensive care unit
IMS: intensive care unit mobility scale
LOS: length of stay
SOFA: the Sequential Organ Failure Assessment
