# Supplementary material for: Impact of age on mortality and transfer to long-term care in patients in an intensive care unit
Source: BMC Geriatr. 2023 Dec 12;23:839. doi: 10.1186/s12877-023-04526-5 (PMC10714659; doi:10.1186/s12877-023-04526-5)
Supplement: Supplementary file 1 — Supplementary Material 1 [file 12877_2023_4526_MOESM1_ESM.docx]

**Supplementary Tables**

**Supplementary Table S1.** Baseline characteristics of participants according to use of facility before admission.

| **Characteristics** | **Non-use of facility before admission group (N =1269)** | **Use of facility before admission group (N = 315)** | **p-value** |
| --- | --- | --- | --- |
| **Age (years)** | 67.0 (57.0–76.0) | 67.0 (58.0–77.0) | 0.927 |
| **Sex, no (%)** |  |  | 0.277 |
| Male, no. | 835 (65.8) | 197 (62.5) |  |
| Female, no. | 434 (34.2) | 118 (37.5) |  |
| **Mean arterial pressure (mmHg)** | 85.0 (72.0–97.0) | 83.0 (69.0–96.0) | 0.025 |
| **Heart rate (/min)** | 100.0 (82.0–118.0) | 97.0 (80.0–113.0) | 0.029 |
| **Respiratory rate (/min)** | 21.0 (18.0–25.0) | 21.0 (17.0–25.0) | 0.925 |
| **BMI (****kg/m^2^)** | 22.5 (19.8–25.3) | 21.7 (18.4–23.7) | <0.001 |
| **ICU admission year, no (%)** |  |  | <0.001 |
| 2017 | 335 (26.4) | 138 (43.8) |  |
| 2018 | 314 (24.7) | 83 (26.3) |  |
| 2019 | 302 (23.8) | 55 (17.5) |  |
| 2020 | 318 (25.1) | 39 (12.4) |  |
| **CCI** | 3.0 (2.0–5.0) | 3.0 (2.0–5.0) | 0.035 |
| **CCI, no (%)** |  |  | 0.163 |
| 0–1 | 238 (18.8) | 74 (23.5) |  |
| 2–4 | 660 (52.0) | 156 (49.5) |  |
| ≥5 | 371 (29.2) | 86 (27.0) |  |
| **Comorbidities, no (%)** |  |  |  |
| HTN | 731 (57.6) | 179 (56.8) | 0.802 |
| DM | 482 (38.0) | 118 (37.5) | 0.864 |
| CKD | 333 (26.2) | 75 (23.8) | 0.377 |
| Cardiovascular disease | 257 (20.3) | 55 (17.5) | 0.265 |
| Cerebrovascular disease | 133 (10.5) | 68 (21.6) | <0.001 |
| Liver disease | 223 (17.6) | 39 (12.4) | 0.026 |
| Asthma | 69 (5.4) | 22 (7.0) | 0.291 |
| COPD | 121 (9.5) | 35 (11.1) | 0.401 |
| Solid cancer | 401 (31.6) | 65 (20.6) | <0.001 |
| Psychological disorder | 23 (1.8) | 9 (2.9) | 0.238 |
| Dementia | 43 (3.4) | 26 (8.3) | <0.001 |
| **Reason for ICU admission** |  |  | <0.001 |
| Cardiogenic cause | 45 (3.5) | 8 (2.5) |  |
| Respiratory cause | 706 (55.6) | 211 (67.0) |  |
| Gastrointestinal cause | 205 (16.2) | 22 (7.0) |  |
| Nephrogenic cause | 94 (7.4) | 14 (4.4) |  |
| Infectious cause | 203 (16.0) | 53 (16.8) |  |
| Hematologic cause | 16 (1.3) | 7 (2.2) |  |
| **ICU LOS (day)** | 8.0 (4.0–17.5) | 11.0 (6.0–19.0) | 0.008 |
| **General ward LOS before ICU admission (day)** | 1.0 (0.0–10.0) | 7.0 (0.0–34.0) | <0.001 |
| **General ward LOS after ICU discharge (day)** | 25.0 (12.0–52.5) | 34.0 (16.0–80.0) | <0.001 |
| **IMS on ICU admission** | 0.0 (0.0–0.0) | 0.0 (0.0–0.0) | 0.755 |
| **IMS on ICU discharge** | 0.0 (0.0–1.0) | 0.0 (0.0–1.0) | 0.151 |
| **DNR status after ICU admission, no (%)** | 471 (37.1) | 114 (36.2) | 0.761 |
| **28-day mortality, no (%)** | 369 (29.1) | 80 (25.4) | 0.194 |
| **90-day mortality, no (%)** | 508 (40.0) | 106 (33.7) | 0.037 |
| **Discharge destination*** |  |  | <0.001 |
| Home | 526 (74.6) | 107 (59.1) |  |
| General hospital | 34 (4.8) | 16 (8.8) |  |
| Long–term care hospital | 145 (20.6) | 58 (32.0) |  |
| **Intubation, no (%)** | 944 (74.4) | 240 (76.2) | 0.510 |
| **SOFA** | 9.0 (6.0–12.0) | 8.0 (6.0–11.0) | 0.014 |
| **Laboratory data** |  |  |  |
| White blood cell (10^3^/µL) | 12.3 (7.5–18.6) | 11.1 (7.6–16.8) | 0.124 |
| Albumin (g/dL) | 2.7 (2.4–3.1) | 2.6 (2.3–2.9) | <0.001 |
| Blood urea nitrogen | 31.5 (19.6–50.7) | 29.0 (18.2–48.1) | 0.082 |
| Creatinine | 1.1 (0.7–1.9) | 0.8 (0.5–1.6) | <0.001 |
| C-reactive protein (mg/L) | 92.5 (33.5–187.8) | 87.0 (37.2–180.9) | 0.995 |
| Delta neutrophil index | 2.6 (0.9–7.8) | 2.4 (0.8–7.2) | 0.413 |

* 886 patients survived and were discharged from the hospital.

BMI, body mass index; CCI, Charlson Comorbidity index; ICU, intensive care unit; LOS, length of stay; IMS, ICU Mobility Scale; DNR, do not resuscitate; SOFA, sequential organ failure assessment score; HTN, hypertension; DM, diabetes mellitus; CKD, chronic kidney disease; COPD, chronic obstructive pulmonary disease

**Supplementary Table S2.** Logistic regression analysis for primary and secondary outcomes in the non-use of facility before admission group.

| Age groups | <65 years | | 65–79 years | | ≥80 years | |
| --- | --- | --- | --- | --- | --- | --- |
|  | 95% CI | p-value | 95% CI | p-value | 95% CI | p-value |
| 28-days mortality, no (%) | 158 / 556 (28.4) |  | 148 / 512 (28.9) |  | 63 / 201 (31.3) |  |
|  |  |  |  |  |  |  |
| OR | 1.0 (ref) |  | 1.02 (0.79–1.34) | 0.860 | 1.15 (0.81–1.63) | 0.434 |
| aOR* | 1.0 (ref) |  | 0.86 (0.62–1.19) | 0.356 | 1.08 (0.70–1.67) | 0.733 |
| 90-days mortality, no (%) | 202 / 556 (36.3) |  | 220 / 512 (43.0) |  | 86 / 201 (42.8) |  |
|  |  |  |  |  |  |  |
| OR | 1.0 (ref) |  | 1.32 (1.03–1.69) | 0.027 | 1.31 (0.94–1.82) | 0.107 |
| aOR* | 1.0 (ref) |  | 1.19 (0.83–1.70) | 0.340 | 1.43 (0.89–2.29) | 0.144 |
| ICU LOS ≥7 days | 309 / 556 (55.6) |  | 308 / 512 (60.2) |  | 131 / 201 (65.2) |  |
|  |  |  |  |  |  |  |
| OR | 1.0 (ref) |  | 1.21 (0.95–1.54) | 0.130 | 1.50 (1.07–2.09) | 0.018 |
| aOR* | 1.0 (ref) |  | 1.21 (0.94–1.54) | 0.137 | 1.55 (1.10–2.17) | 0.012 |
| Discharge to home† | 270 / 325 (83.1) |  | 195 / 268 (72.8) |  | 61 / 112 (54.5) |  |
|  |  |  |  |  |  |  |
| OR | 1.0 (ref) |  | 0.54 (0.37–0.81) | 0.003 | 0.24 (0.15–0.39) | <0.001 |
| aOR* | 1.0 (ref) |  | 0.54 (0.35–0.81) | 0.003 | 0.24 (0.14–0.40) | <0.001 |
| Discharge to general hospital † | 17 / 325 (5.2) |  | 9 / 268 (3.4) |  | 8 / 112 (7.1) |  |
|  |  |  |  |  |  |  |
| OR | 1.0 (ref) |  | 0.63 (0.28–1.44) | 0.271 | 1.39 (0.58–3.32) | 0.454 |
| aOR* | 1.0 (ref) |  | 0.73 (0.30–1.77) | 0.484 | 1.59 (0.63–4.02) | 0.324 |
| Discharge to long–term care hospital † | 38 / 325 (11.7) |  | 64 / 268 (23.9) |  | 43 / 112 (38.4) |  |
|  |  |  |  |  |  |  |
| OR | 1.0 (ref) |  | 2.37 (1.53–3.68) | <0.001 | 4.71 (2.83–7.83) | <0.001 |
| aOR* | 1.0 (ref) |  | 2.30 (1.46–3.62) | <0.001 | 4.56 (2.65–7.85) | <0.001 |

* Adjusted for sex, BMI, CCI, SOFA, general ward LOS before ICU admission, and DNR status after ICU admission.

† 705 patients who survived.

BMI, body mass index; CCI, Charlson comorbidity index; SOFA, sequential organ failure assessment score; aOR, adjusted odds ratio; CI, confidence interval

**Supplementary Table S3.** Logistic regression analysis for primary and secondary outcomes in the use of facility before admission group.

| Age groups | <65 years | | 65–79 years | | ≥80 years | |
| --- | --- | --- | --- | --- | --- | --- |
|  | 95% CI | p-value | 95% CI | p-value | 95% CI | p-value |
| 28-days mortality, no (%) | 30 / 138 (21.7) |  | 36 / 131 (27.5) |  | 14 / 46 (30.4) |  |
|  |  |  |  |  |  |  |
| OR | 1.0 (ref) |  | 1.36 (0.78–2.38) | 0.275 | 1.58 (0.75–3.32) | 0.233 |
| aOR* | 1.0 (ref) |  | 0.92 (0.46–1.85) | 0.823 | 0.91 (0.36–2.30) | 0.840 |
| 90-days mortality, no (%) | 42 / 138 (30.4) |  | 48 / 131 (36.6) |  | 16 / 46 (34.8) |  |
|  |  |  |  |  |  |  |
| OR | 1.0 (ref) |  | 1.32 (0.80–2.20) | 0.281 | 1.22 (0.60–2.47) | 0.583 |
| aOR* | 1.0 (ref) |  | 0.81 (0.41–1.62) | 0.553 | 0.54 (0.21–1.40) | 0.202 |
| ICU LOS ≥7 days | 89 / 138 (64.5) |  | 94 / 131 (71.8) |  | 40 / 46 (87.0) |  |
|  |  |  |  |  |  |  |
| OR | 1.0 (ref) |  | 1.40 (0.84–2.34) | 0.202 | 3.67 (1.45–9.27) | 0.006 |
| aOR* | 1.0 (ref) |  | 1.28 (0.75–2.21) | 0.366 | 3.31 (1.27–8.64) | 0.014 |
| Discharge to home† | 64 / 85 (75.3) |  | 33 / 71 (46.5) |  | 10 / 25 (40.0) |  |
|  |  |  |  |  |  |  |
| OR | 1.0 (ref) |  | 0.29 (0.15–0.56) | <0.001 | 0.22 (0.09–0.56) | 0.002 |
| aOR* | 1.0 (ref) |  | 0.32 (0.15–0.66) | 0.002 | 0.24 (0.07–0.87) | 0.030 |
| Discharge to general hospital † | 6 / 85 (7.1) |  | 6 / 71 (8.5) |  | 4 / 25 (16.0) |  |
|  |  |  |  |  |  |  |
| OR | 1.0 (ref) |  | 1.22 (0.37–3.95) | 0.746 | 2.51 (0.65–9.71) | 0.183 |
| aOR* | 1.0 (ref) |  | 1.06 (0.27–4.09) | 0.938 | 0.22 (0.41–11.96) | 0.355 |
| Discharge to long–term care hospital † | 15 / 85 (17.6) |  | 32 / 71 (45.1) |  | 11 / 25 (44.0) |  |
|  |  |  |  |  |  |  |
| OR | 1.0 (ref) |  | 3.83 (1.85–7.93) | <0.001 | 3.67 (1.40–9.64) | 0.008 |
| aOR* | 1.0 (ref) |  | 3.49 (1.62–7.49) | 0.001 | 3.52 (1.04–11.91) | 0.043 |

* Adjusted for sex, BMI, CCI, SOFA, general ward LOS before ICU admission, and DNR status after ICU admission.

† 181 patients who survived.

BMI, body mass index; CCI, Charlson comorbidity index; SOFA, sequential organ failure assessment score; aOR, adjusted odds ratio; CI, confidence interval
